# 

**Published:** 1914-08-01

**Authors:** 


### CONTENTS.

| | PAGE |
|---|---|
| **The Decay of Moral Force** ... ... ... | 481 |
| **The Visit of North American Surgeons** ... | 482 |
| **Hospital and Institutional News** ... ... ... | 483 |

The Water Famine at Wolverhampton—Complaints against a Wandsworth Infirmary—Over Thirty Years Secretary and Superintendent—New Secretary of the London Lock Hospital—The Value of the Uniform System—The Medical Services of Bradford—Guardians and the Mentally Deficient—Early Mental Cases in Melbourne—A Subscriber's Enthusiasm at Bristol—Pay-Wards at the New Hospitals—Asylums Board Staff and Union Badges—A New Poor-Law Infirmary for Derby—Staff Resignations at Barry Hospital—The Proceeds of Alexandra Day—Suggested New Hospital for Blackpool—"Secret Remedies" Vindicated—A Surrey Sanatorium for Women—Panel Doctors and Disclosed Names—A Long Casualty List—A Hospital President's Warning—The London Ambulance Service—The Home for Epileptics, Maghull—The Isolation Problem at Bristol—A Cottage Hospital for Harwich—This Week's Drug Market.

| | PAGE |
|---|---|
| **The Electrical and Radio-Therapeutic Department of the Cancer Hospital (Free), London** | 489 |
| **Surgeons and Nurses** ... ... ... ... | 492 |
| A Change in the Former's Behaviour. | |
| **Medical Appointments** ... ... ... ... | 492 |

### CONTENTS—(*continued*).

PAGE

**Reports on Hospitals of the United Kingdom**— 493
By SIR HENRY BURDETT, K.C.B., K.C.V.O.
Three Leicester Hospitals: (1) The Faire Hospital—(2) The Maternity Hospital—(3) The Highfield Hospital.

**East Suffolk and Ipswich Hospital** ... ... 494

**National Insurance Act Developments** ... ... 495

The New Insurance Books.

**Institutional Organisation in America** ... 497

III. Reforms in Gynæcological Out-Patient Clinics.

**Research and Remedies** ... ... ... ... 498

**The Bristol General Hospital** ... ... ... 499
Opening of a Large New Wing.
Architects' Description of the Buildings.

**Liverpool Royal Infirmary** ... ... ... ... 501
Opening of a New Ward and Laboratory for Tropical Diseases.

**Round the World** ... ... ... ... ... ... 502

Fellow-Workers and their Doings.

PAGE

**Early Treatment of Mental Cases** ... ... 503
Proposed Use for Reception Homes and Observation Wards.

**The Editor's Letter-Box** ... ... ... ... 504
Employers' Subscriptions and Compensation Cases—The Secretaryship of St. Mark's—The Royal Portsmouth Hospital.

**The Profession of Medicine** ... ... ... 506
The Clinical Congress of Surgeons of North America.
The British Medical Association Meeting.

**The Institutional Worker** ... ... (Suppl.) 1
Points in Institutional Housekeeping.
Questions for August and September.
Questions Invited.
Employment and Training.
Miscellaneous.
The Sick and in Need.
Practical Matters.
Acknowledgments.

**Buildings Contemplated** ... ... (Suppl.) 3

**The Book Market** ... ... ... ,, 3

